# Metformin: Targeting the Metabolo-Epigenetic Link in Cancer Biology

**DOI:** 10.3389/fonc.2020.620641

**Published:** 2021-02-02

**Authors:** Elisabet Cuyàs, Sara Verdura, Begoña Martin-Castillo, Javier A. Menendez

**Affiliations:** ^1^ Girona Biomedical Research Institute, Girona, Spain; ^2^ Program Against Cancer Therapeutic Resistance (ProCURE), Metabolism & Cancer Group, Catalan Institute of Oncology, Girona, Spain; ^3^ Unit of Clinical Research, Catalan Institute of Oncology, Girona, Spain

**Keywords:** metformin, metabolism, epigenetics, chromatin, cancer, diet, microbiota

## Abstract

Metabolism can directly drive or indirectly enable an aberrant chromatin state of cancer cells. The physiological and molecular principles of the metabolic link to epigenetics provide a basis for pharmacological modulation with the anti-diabetic biguanide metformin. Here, we briefly review how metabolite-derived chromatin modifications and the metabolo-epigenetic machinery itself are both amenable to modification by metformin in a local and a systemic manner. First, we consider the capacity of metformin to target global metabolic pathways or specific metabolic enzymes producing chromatin-modifying metabolites. Second, we examine its ability to directly or indirectly fine-tune the activation status of chromatin-modifying enzymes. Third, we envision how the interaction between metformin, diet and gut microbiota might systemically regulate the metabolic inputs to chromatin. Experimental and clinical validation of metformin’s capacity to change the functional outcomes of the metabolo-epigenetic link could offer a proof-of-concept to therapeutically test the metabolic adjustability of the epigenomic landscape of cancer.

## Introduction

Global changes in the topology of the epigenetic landscape are a hallmark of cancer (1–3). Metabolism can directly drive or indirectly enable an aberrant chromatin state of cancer cells (4–9). Elucidating pharmacological and dietary interventions that can fine-tune the molecular cross-talk between metabolism and the chromatin state is an active and rapidly growing area in cancer research (10–12).

**Figure 1:**
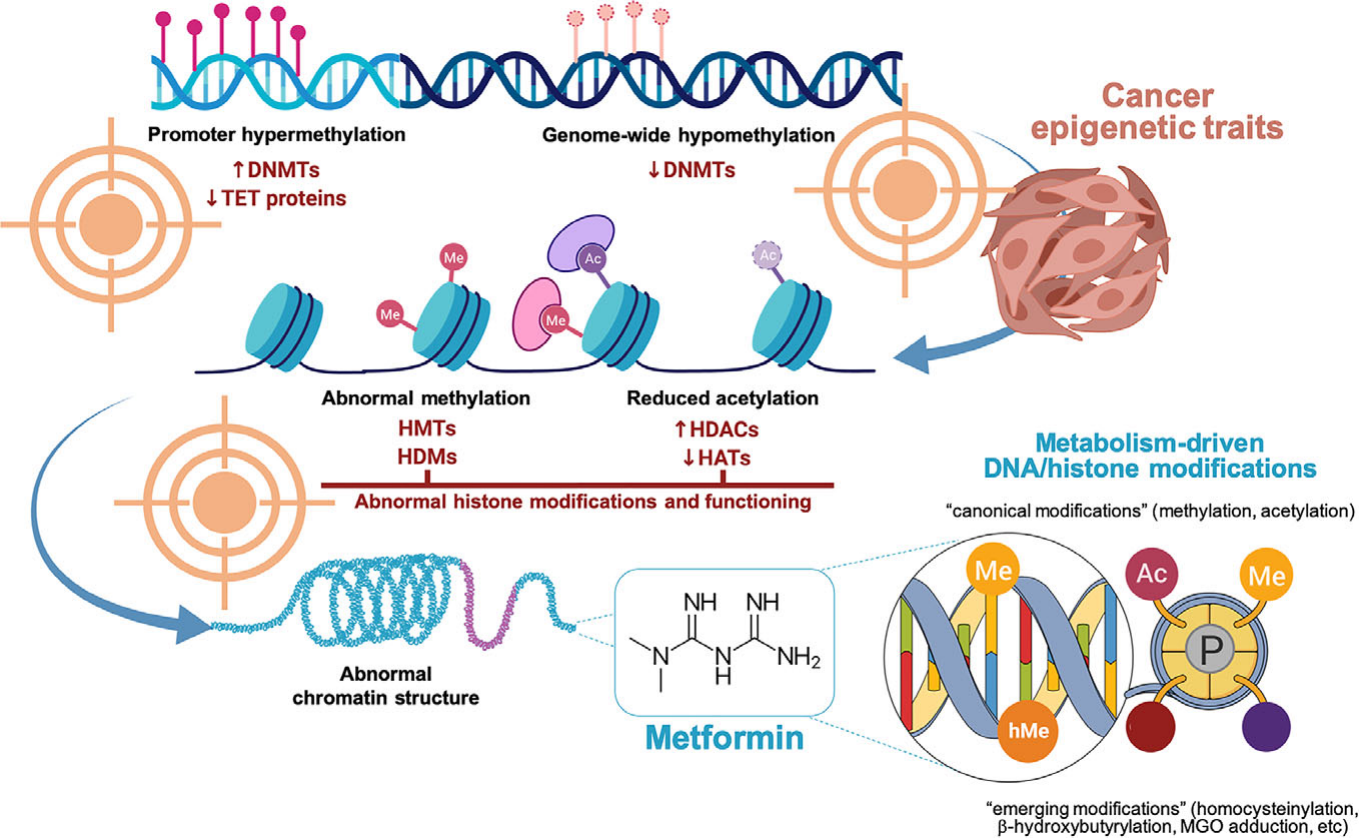
Metformin and the epigenetic landscape in cancer biology. The epigenetic traits in human cancers may lead to at least four five major phenotypes, namely: DNA promoter hypermethylation, genome-wide DNA hypomethylation, abnormal histone modifications (e.g., acetylation, methylation), and abnormal chromatin structure. We are accumulating ever-growing evidence that metformin can reverse several of the metabolism-related DNA/histone modifications underlying cancer biology including abnormal DNA methylation patterns, reduced histone acetylation, and abnormal histone methylation marking. It remains unclear whether and how metformin could influence the high-level architecture of chromosomes. Created with BioRender.com.

More than 60 years after its introduction as a first-line therapy for managing type 2 diabetes, the biguanide metformin is emerging as an archetypal drug capable of targeting most of the epigenetic traits of cancer (13–16) (**Figure 1**). The role of metformin as a remodeler of the epigenetic landscape largely relies on its multi-faceted capacity to link metabolic signals to chromatin status. The ability of metformin to modify the functional outcomes of the metabolo-epigenetic link might serve as a proof-of-concept to therapeutically test the metabolic adjustability of the epigenomic landscape in cancer biology. Here, we briefly review how the biochemical principles of metabolite-derived chromatin modifications and the metabolo-epigenetic machinery itself are both amenable to regulation by metformin in a local and systemic manner.

## Metformin and Chromatin-Modifying Metabolites

**Figure 2:**
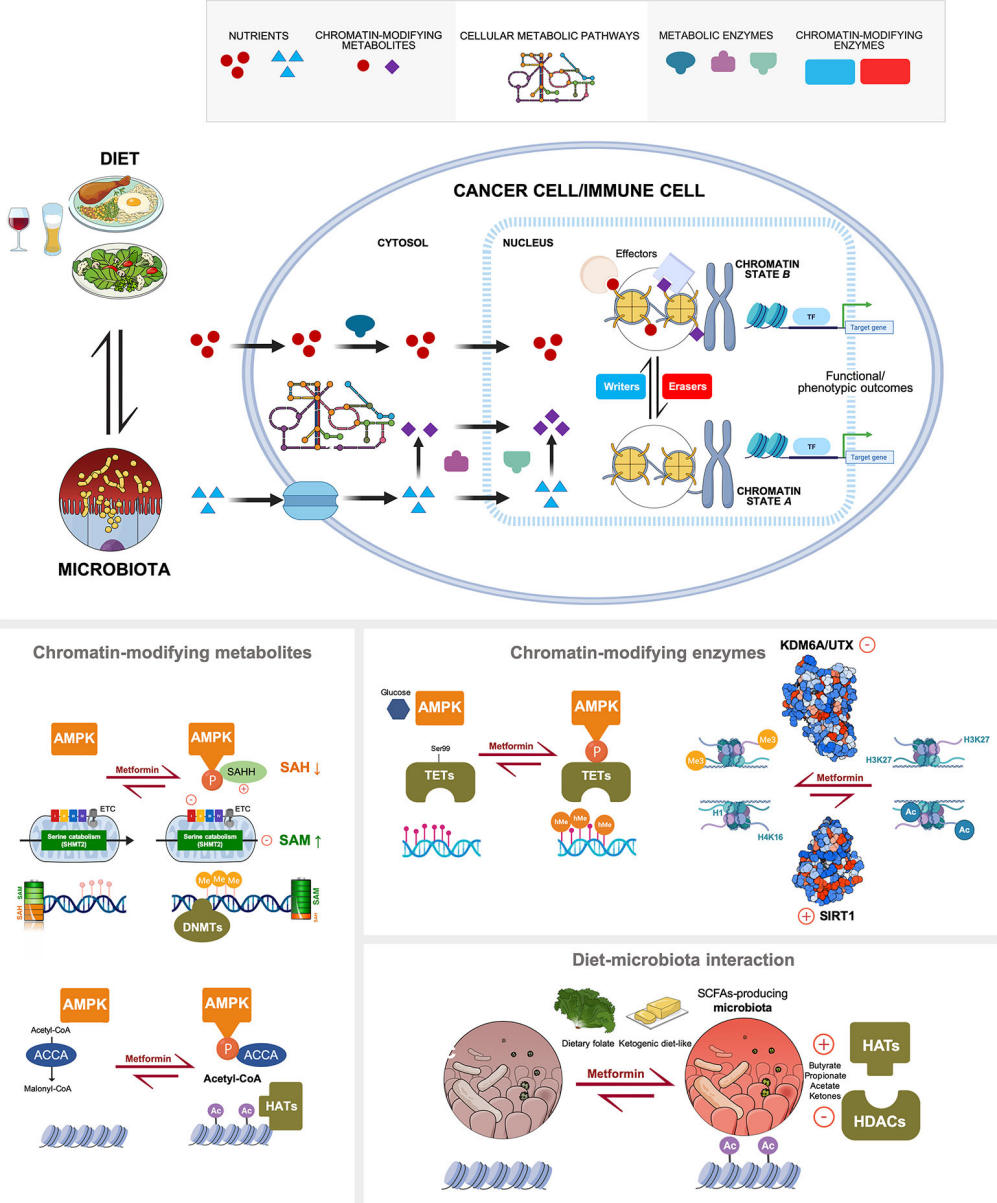
Multi-layered regulation of the cancer metabolo-epigenetic link by metformin. *Top.* Nutrients and metabolites derived from the bidirectional interaction between diet and human microbiome are directly utilized or internally processed by cellular metabolic pathways (either globally in the cytosol or locally in the nucleus) to modify chromatin. The catalytic dependence of chromatin-modifying enzymes on the abundance of substrates or activity modulators (cofactors) dictates, at least in part, both the type and rate of chromatin modifications. The metabolic regulation of epigenetic markings lastly determine the expression of genes related to functional outcomes (e.g., stemness/differentiation, immune suppression/activation, etc) that are relevant not only at the cancer cell-autonomous level but also to a variety of immune cell types. *Bottom.* All the physiological and molecular principles of the local and systemic metabolic link to cancer epigenetics can be targeted with the anti-diabetic biguanide metformin, namely: global metabolic pathways or specific metabolic enzymes producing chromatin-modifying metabolites, activation status of chromatin-modifying enzymes, and the interaction between diet and gut microbiota. Created with BioRender.com.

Many cellular metabolic pathways related to central-carbon/tricarboxylic acid cycle, one-carbon/methionine metabolism, acetate metabolism, ketogenesis, and redox balance produce metabolites that are used as substrates and cofactors by chromatin-modifying enzymes (4–12). The physiological concentrations of these metabolites are commensurate with the catalytic efficiency of some chromatin-modifying enzymes (e.g., methyltransferases and acetyltransferases, among many others). Perturbations in the abundance of chromatin-modifying metabolites can therefore influence the activities of certain chromatin-remodeling enzymes and, consequently, the types and/or levels of “classical” (methylation, acetylation) and “emerging” (e.g., lactylation, butyrylation, homocysteinylation, etc) epigenetic modifications (4–8, 12). Metformin can fine-tune the relative sensitivity of epigenetic regulators to metabolic alterations by modifying the functioning of global metabolic pathways or specific metabolic enzymes producing chromatin-modifying metabolites (**Figure 2**).

Metformin-driven changes in the metabolic pathways producing chromatin-modifying metabolites can be orchestrated in parallel with mitochondrial complex I (mCI) inhibition and its inhibitory action on the mitochondrial electron transport chain (ETC), and can be attributed both to its AMP-activated protein kinase (AMPK)-dependent and -independent actions. There are numerous examples that illustrate the ability of metformin to alter the pools of metabolic intermediates acting as activity modulators of a variety of epigenome-modifying enzymes, including nicotinamide adenine dinucleotide (NAD^+^) and β-hydroxyglutarate for histone deacetylases (HDACs), acetyl-CoA for histone acetyltransferases (HATs), α-ketoglutarate/succinate for histone/DNA demethylases (HDMs), and s-adenosyl-methionine/homocysteine (SAM/SAH) for DNA/histone methyltransferases (DNMTs/HMTs), among others (15, 17–28). The ability of metformin to tweak the SAM : SAH ratio-dependent methylation capacity exemplifies how it can regulate molecular inputs to chromatin *via* AMPK-dependent and -independent regulatory actions on central epigenetic metabolites. Metformin-induced AMPK activation positively modulates the activity of SAH hydrolase (SAHH) (29), to decrease the availability of SAH, a potent feedback inhibitor of SAM-dependent DNA and histone methyltransferases. In an AMPK-independent manner, the metformin-driven disruption of the coupling between mCI activity and serine hydroxymethytransferase (SHMT2)-driven mitochondrial serine catabolism increases the contribution of one-carbon units to SAM from folate stores (30). The targeting of core “hub” metabolites with epigenetic properties, such as SAM and SAH, supports the notion that metformin can activate epigenetic cancer-protection programs by promoting genome-wide DNA methylation events including methylation-regulated motility of retrotransposable elements such as long interspersed nuclear element-1 (LINE-1) or hypermethylation of tumor-promoting pathway genes (15, 29–31). Yet, we should acknowledge that there is a paucity of mechanistic studies examining a causal relationship between metformin-induced alterations in specific metabolites, regulation of the epigenomic landscape in concert with metabolite-associated chromatin modifiers, and epigenetic control of cancer-related functional outcomes.

## Metformin and Chromatin-Modifying Enzymes

Beyond promoting fluctuations in the abundance of metabolites that can positively or negatively influence the activities of certain chromatin-modifying enzymes, metformin can modulate the levels of specific epigenetic modifications by indirectly or directly targeting the activation status of core epigenetic enzymes (**Figure 2**).

Perhaps the most obvious way in which metformin can target epigenetic enzymes is *via* the multifunctional kinase effects of AMPK (32–35). By inhibiting mCI and oxidative phosphorylation, metformin increases the AMP : ATP ratio, which induces the activation of AMPK. In turn, AMPK can directly regulate the phosphorylation/activation status of some DNA methyltransferases isoforms (e.g., DNMT1), which catalyze the methylation of cytosine-guanine dinucleotides (CpG), in addition to the ten-eleven translocation hydroxylases (TET), which catalyze the conversion of 5-methylcytosine to unmethylated cytosine. AMPK activation also induces the phosphorylation of histones/histone-modifying enzymes that play a major role in the regulation of the structure and function of nucleosomes (36), namely: histone H2B to promote chromatin relaxation, HDACs to increase histone acetylation, polycomb repressive complex 2 (PRC2) to decrease histone methylation, poly [ADP-ribose] polymerase 1 (PARP1) to increase histone ribosylation, and *O-*linked β-N-acetylglucosamine (*O*-GlcNAc) transferase to inhibit histone *O*-GlcNAcylation. There are already some encouraging examples of how metformin-induced AMPK activation regulates the activity of DNA methyltransferases/demethylases and histone-modifying enzymes to translate the metformin-driven transient decrease in cellular energy status into epigenetically-regulated transcriptional events. At the level of DNA methylation, metformin can protect the AMPK-mediated phosphorylation of the TET2 demethylating enzyme, which increases TET2 stability and 5-hydroxymethylcytosine (5 hmC) levels, thus impeding the ability of certain metabolic environments (e.g., high glucose state) to aberrantly reprogram the epigenome, thereby maintaining genomic stability (37). At the level of histone post-translational modifications, metformin-induced AMPK activation has been shown to phosphorylate the histone methyltransferase EZH2 (enhancer of zeste homolog 2), leading to the attenuated methyltransferase activity (and oncogenic function) of PRC2 (polycomb repressive complex 2) (38).

One should acknowledge that the aforementioned ability of metformin to regulate the substrate/cofactor availability of chromatin-modifying metabolites adds an additional layer of regulatory complexity to its capacity to drive histone modifications. For instance, metformin treatment reduces intracellular succinate levels and activates AMPK, both of which can independently mediate the activation of the lysine-demethylase 2 A (KDM2A) to repress ribosomal RNA (rRNA) transcription and cell proliferation (24). Moreover, certain epigenetic enzymes appear to behave as metformin-binding proteins, pointing to the possibility that metformin can directly modulate their chromatin-modifying activities without the involvement of mCI and AMPK. Metformin-induced activation of AMPK can indirectly activate the class III HDAC SIRT1 (Sirtuin 1) by increasing the NAD^+^/NADH ratio (17, 39, 40). Intriguingly, the AMPK-independent behavior of metformin as a *bona fide* SIRT1 agonist might also rely on its ability to directly improve the catalytic efficiency of SIRT1 operating under conditions of low NAD^+^ (41). Although metformin-induced inhibition of catalysis by isolated mCI, which is believed to be the primary target of metformin, requires concentrations as high as 20–100 mmol/L (42, 43), the physiological relevance of the direct binding of metformin to chromatin modifiers such as SIRT1 (41) and KDM6A/UTX (44) at millimolar concentrations in excess of the therapeutic levels achieved in human patients remains unknown.

## Metformin and Diet/Microbiota-Associated Epigenetic Reprogramming

Environmental factors including diet and microbiota can regulate HMTs and HATs by modulating the intracellular pools of their substrates SAM and acetyl-CoA, respectively. Dietary methionine and other nutrients feeding into one-carbon and methionine metabolism (e.g., folate, vitamin B, and choline) can modulate the levels of SAM and SAH to reprogram the landscape of DNA and histone methylation (10, 45–47). Metabolic adaptation to calorie restriction, fasting, the intake of a ketogenic diet, and exercise, can generate ketone bodies through the breakdown of fatty acids and ketogenic amino acids (48–54). The ketone body β-hydroxybutyrate (β-OHB) can elicit multi-faceted chromatin modifications by inhibiting class I HDACs to cause global upregulation of histone acetylation (55), while also serving as a substrate for β-hydroxybutyrylation, which associates with the upregulation of starvation-responsive metabolic pathways (56). The ketone body acetoacetate can suppress the adduction of histones by methyglyoxal (MGO) (57), a reactive dicarbonyl sugar metabolite that induces disease-related changes in chromatin architecture and the epigenetic landscape (58, 59).

The bidirectional interaction between diet and gut microbiota can drive microbiota metabolomes to alter systemic metabolic physiology and influence epigenetic programs (60–62) (**Figure 2**). Microbiotal activity on dietary fiber can enhance circulating levels of short-chain fatty acids (SCFAs) such as a formate [C1], acetate [C2], propionate [C3], butyrate/isobutyrate [C4], crotonate [C4], and valerate/isovalerate [C5], which can feed the intracellular pool of acetyl-CoA and/or promote the inhibition of HDACs to augment histone acetylation and crotonylation (63–66). Lesser-explored diet/gut microbiota interactions might involve the colonization of specific microbiota species competing with host cells for epigenome metabolites, such as choline-consuming strains reducing the serum levels of the methyl donor SAM, and consequently promoting changes to global DNA methylation (67). Formaldehyde, a formate-related one-carbon benign/genotoxic molecule at the crossroad between cellular and whole-body metabolism, coupled with the societal use of alcohol consumption (68, 69), might alter epigenetic regulation as a source of pathological formyl-lysine that is refractory to HDACs (70, 71).

Early evidence of the role of metformin in modulating the gut microbiota involved the modulation of microbial folate, leading to methionine restriction in *C. elegans* (72). The addition of metformin to an established anti-breast cancer therapeutic regimen was found to induce a fasting- and antifolate-modification of systemic host metabolism that included a significant increase in the circulating levels of the ketone body β-OHB and of homocysteine (28, 73), a surrogate marker of the availability of methyl groups (SAM) *via* one-carbon/methionine metabolism. In diabetic individuals, metformin can also shift gut microbiota composition through the enrichment of predominantly SCFA-producing bacteria (74, 75). Metformin is also known to significantly reduce systemic MGO levels, not only by operating as an efficient scavenger (76) but by also by increasing the activity of glyoxalase 1, the major route of MGO detoxification (77). The ability of metformin to fine-tune the shaping influence of environmental factors on the epigenomic landscape might go beyond the bidirectional cross-talk between diet and microbiota (**Figure 2**). In this regard, the reduction of circulating formate as a consequence of metformin-induced disruption of the coupling between mCI activity and mitochondrial serine catabolism (78, 79) and the recently described formaldehyde-scavenging capacity of metformin (80), add new metabolic facets likely involved in the systemic effects of metformin on the cancer-driving metabolo-epigenetic link. Yet, it remains an open question whether systemic metabolic adaptation to metformin through modulation of methionine/one-carbon metabolism, which might be secondary to reduced vitamin B12 and/or folate levels (81), or *via* nutritional modulation of the gut microbiota [e.g., decreasing the availability of branched-chain amino acids (82)], suffices to induce distinct epigenetic responses capable of regulating cancer-relevant phenotypic outcomes.

## Metformin-Targeted Metabolo-Epigenetics Axis in Cancer Prevention and Treatment: Are We There Yet?

We are accumulating more and more evidence that metformin can dynamically reshape the epigenomic landscape of cancer. First, metformin can target global metabolic pathways or specific metabolic enzymes producing chromatin-modifying metabolites. Second, metformin can indirectly or directly modify the activation status of chromatin-modifying enzymes. Third, metformin can interact with diet/gut microbiota to systemically regulate the metabolic inputs to chromatin. However, despite the exciting progress in deciphering how metformin influences metabolically regulated epigenetic marks, we should acknowledge that mechanistic understanding of how it can reverse cancer-relevant functional outcomes [e.g., phenotypic plasticity and cell fate decisions in heterogenous cancer populations, metabolism-epigenome axes controlling anti-tumor immunity and immunotherapy efficacy (83–87)] by interfering with the metabolic regulation of the epigenome remains unsatisfactory.

Important future questions for the field include: Can metformin impact the distribution of metabolites between the nuclear and non-nuclear compartment by changing metabolite consumption on and release from chromatin (88)? Can metformin reconduct the same core metabolic pathways or metabolites to establish distinct epigenetic signatures of the heterogenous tumor microenvironment at the single cell level (89)? Can metabolic regulation of higher-order genomic organizers elicit a metformin-driven regulation of the three-dimensional architecture of chromosomes (90–92)? These remain exciting areas of investigation. While decoding the molecular intimacy of the metabolo-epigenetic “language” of metformin in cancer biology, it remains true that the experimental and clinical validation of its capacity to alter the functional outcomes of the metabolo-epigenetic link could provide a proof-of-concept to therapeutically test the metabolic adjustability of the epigenomic landscape in cancer prevention and treatment.

## Author Contributions

JM conceived the scope of the manuscript, as well as for drafting the manuscript. EC, SV, and BM-C helped with final formatting of the manuscript, schematics and literature search and selection. All authors contributed to the article and approved the submitted version.

## Funding

Work in the Menendez laboratory is supported by the Spanish Ministry of Science and Innovation (grants SAF2016-80639-P and PID2019-10455GB-I00, Plan Nacional de l+D+I, founded by the European Regional Development Fund, Spain) and by an unrestricted research grant from the Fundació Oncolliga Girona (Lliga catalana d’ajuda al malalt de càncer, Girona). EC is a recipient of a research contract “Miguel Servet” (CP20/00003) from the Instituto de Salud Carlos III, Spanish Ministry of Science and Innovation (Spain).

## Conflict of Interest

The authors declare that the research was conducted in the absence of any commercial or financial relationships that could be construed as a potential conflict of interest.
